# Active Consideration of Future Health Can Be Prompted by Simple Health Messages and Improves Nutritional Quality of Food Choices

**DOI:** 10.3389/fnut.2022.926643

**Published:** 2022-07-07

**Authors:** Christopher R. Gustafson

**Affiliations:** Department of Agricultural Economics, University of Nebraska-Lincoln, Lincoln, NE, United States

**Keywords:** future consideration, food choice, nutrition, point of decision prompt, choice process variables, dietary fiber, attention

## Abstract

Many choices that people face daily have implications for future health and well-being. Choices about what foods to purchase and consume are one of the most frequent—and universal choices—that people must make. The ongoing rise of overweight and obesity rates—and associated diet-related diseases—in the US and many other countries illustrates the future health consequences of low-quality dietary choices. While a large body of research shows that individuals with a tendency to consider the future make a wide range of healthier decisions, research on limited attention and exogenous factors influencing choice suggests that attention to the future consequences of choices may vary from one choice scenario to the next. In this research, we examine the impact of active consideration of future health impacts during a hypothetical online food choice experiment on the nutritional quality of food choices and on choice process variables—the set of products people choose to select from and the use of nutrition information during choice—during an online food choice task. Next, we examine the impact of exposure to a short message about the health benefits of fiber on consideration of future health impacts and on the nutritional quality of choices. We find that active consideration of future health impacts significantly improves the nutritional quality of choices—particularly among processed food products—and makes people more likely to pay attention to healthy foods and use nutrition information. Exposure to a short health message significantly increases the likelihood that individuals consider future health impacts during choice, which promotes healthier choices overall.

## Introduction

Low savings rates; health problems related to poor diet and low levels of physical activity (among others); and under-investment in human capital are all behaviors exhibited by significant portions of the public that carry potentially dire consequences for those individuals (1–3). A common element of all these decisions is that the decision an individual makes now has implications for their future. Spending money now may satisfy the individual's desire for a material good but prevents the individual from having the money available to use later.

Preference-based models of intertemporal choice suggest that people make these choices by weighing the benefits and costs of different options occurring at different points of time based on a rate at which they discount the future (4). Discount rates may be consistent across time or biased toward immediate benefits, but in all cases, the decision-maker considers the current and future impacts of choices. However, recent research suggests that people may not consistently pay attention to the implications of choices across time. Read et al. (5) find that in intertemporal choice tasks in which individuals choose between a smaller, earlier amount of money or a larger, delayed amount, people asymmetrically consider opportunity costs—the opportunities they forego when choosing a specific option—which affects the choices they make. Specifically, they document that people consider immediate opportunity costs—the benefits they give up now to wait for a reward—but are less likely to consider the opportunity costs they forego in the future to obtain the immediate benefit.

### Asymmetric Attention to Opportunity Costs and Future Orientation

Blindness to future opportunity costs has important implications for many common decisions. These include decisions about purchases now, preventing the use of those funds later—which represents a future opportunity cost—as well as food choices, where choosing a favored, but oftentimes less healthy, item today has a future health (opportunity) cost. Various scales—predominantly in psychology—have been developed to capture individuals' tendencies to think about the future, or future orientation. A meta-analysis of studies using measures of future orientation finds that individuals who are future oriented are more likely to engage in a host of behaviors that benefit the individual in the long-run, including physical activity, retirement savings, and human capital accumulation (6). Many of these constructs, such as the consideration of future consequences scale, are intended to capture an underlying personality trait that is stable within an individual across time (7). Longitudinal research suggests that trait stability is a reasonable assumption for shorter periods of time, but that over multiple years future orientation measures may change (8). Further, experimental evidence suggests that future orientation is causally related to healthy choices and beneficial outcomes (9).

### External Influences on Attention in Decision-Making

While the links between consideration of the future and decisions that will be healthier for the individual in the long term are robust, there is also strong evidence that cognitive processes involved in decision-making can be affected by external factors, which may lead to variation in an individual considering the future implications of the choice alternatives they face from one instance of choice to the next. For instance, individuals with low incomes who were exposed to a hypothetical scenario describing an unanticipated, higher cost car repair performed worse on cognitive tests than low-income individuals who were exposed to a scenario about unanticipated but lower costs repairs or high-income individuals who were exposed to either lower or higher cost hypothetical repairs (10). People exposed to survey questions about overdraft fees were less likely to incur fees during the survey month (11). Exposure to questions about overdraft fees on multiple surveys led to decreased risk of overdraft fees over multiple years (11), suggesting that increased attention to behaviors that put people at risk of those fees changes behaviors in protective ways. Stock prices have been found to be influenced by the presence of sunshine during the morning hours of a trading day, presumably by making traders more optimistic (12). On the other hand, sadness, which, like other emotions, can be systematically affected by external factors (13), has been shown to levy financial costs by affecting people's decisions (14). In the domain of food choice, hunger has been shown to have effects on foods chosen for both immediate and future consumption (15).

Evidence that these external factors affect decision-making processes suggests that while individuals may differ in their general tendency to consider the future, there is likely to be situation-specific variation in attention to the broader implications of choices beyond the general tendency. Situation-specific variation in the consideration of future consequences may provide an opportunity to implement interventions aimed at priming or prompting individuals to consider the broader implications of the choices they face (16, 17). This may be particularly true in complex decision-settings in which people have to process numerous options in order to make a choice. Research shows that increases in the number of items or attributes in a choice set reduces attention to attribute information (18, 19), which could inhibit the consideration of long-term impacts of variation in those unconsidered attributes.

### Situation-Specific Consideration of Future Impacts During Choice: A Research Plan

This research examines active consideration of future health impacts of food consumption in a large sample that is nationally representative by important demographic characteristics during a food choice task in a complex food choice environment, reflective of supermarkets. Consumers face a vast array of products and product categories in typical food retail outlets—a typical well-stocked supermarket has tens of thousands of products, with many individual product categories containing hundreds of unique products (20–22). With such a large number of products to select from, most consumers will not consider all available products. Instead, consumers form a *consideration set*—a small set of alternatives that typically contains 2-5 products that the individual actively considers and selects from (23–25). Food choices tend to be highly habitual (26, 27), which may decrease the likelihood that broader impacts are actively considered during choice processes.

I analyze the relationship of future consideration to the nutritional quality of the foods participants selected in the food choice task. Importantly, unlike a stable individual-specific tendency to consider future consequences of choices, understanding choice-specific consideration offers novel possibilities to intervene during the choice process. While an intervention tested by Hall and Fong (9) delivered information about longer term thinking through three half-hour sessions across multiple weeks, intervening in scenario-specific long-term consideration patterns may require only that the information be provided at a temporally strategic point that influences the decision-maker's cognitive processes—such as just prior to the point of decision. Point of decision prompts have been shown to be effective at promoting healthier food choices and increased physical activity (17, 28); the impact of these prompts on cognition have not been studied.

This paper reports the relationship between active consideration of future health impacts of the foods participants face on the healthiness of the consideration set participants chose to examine, their use of nutrition information during choice, and the nutritional quality of foods chosen in a no-information control group. Then, the research examines the impact of a health prompt on consideration of future health impacts to investigate whether interventions can promote healthier choices by drawing attention to future consequences of the choices people face.

## Materials and Methods

Data were collected from 4622 residents who were ≥19 years of age of the United States via a survey-based food choice experiment and questionnaire. The data were collected between June 11 and July 1, 2021. Surveys were distributed by IRi (www.iriworldwide.com) to a sample that approximates the composition of the US population by gender, age, and income. Approximately half (*n* = 2209) of the participants completed a control condition of the survey. These responses are used for the initial analyses examining the relationship between consideration of future health impacts and choices. The other approximately half of the sample (*n* = 2213) was exposed to a short prompt message about the health benefits of dietary fiber consumption which is an under-consumed and infrequently considered nutrient of public health concern in the US (29, 30), immediately before making food choices. These data are combined with data from the control condition to examine the impact of a simple prompt message on the likelihood of considering the future during food choice and on choice outcomes.

In the survey, respondents completed a hypothetical food choice task first, followed by choice-related questions (e.g., whether respondents used nutrition information that was listed under each food item in the food choice task) and standard demographic questions (e.g., the respondents' gender, age, education, and income). Importantly for this research, one question asked participants to indicate broader implications of the choice that they had considered during the choice process from a list of options. The key option was “Impacts the foods might have on your/your family's health in the future;” other options were included as decoy options to prevent respondents from intuiting that consideration of health was the focus of the study, which might generate an experimenter demand effect (31). Other questions asked participants to indicate whether they used nutrition information that was available below pictures of the items during the choice process. Prices for the food products were based on current prices of those products at a national supermarket chain. A cheap talk script was used to encourage respondents to weigh the real-world uses of the money they would (hypothetically) spend on the items in the food choice task. Cheap talk scripts have been found to reduce the impact of hypothetical bias on choices (32).

### Food Choice Task

The first component of the research involved hypothetical food choices from five food categories. However, participants were asked to make the choices as though they were real and were encouraged to think about other ways in which they might use their money, which is known as a cheap-talk script and has been widely used to address biases from hypothetical choice (32). Specifically, the instructions to participants were, “*When making your food selections, imagine that you are doing a normal shopping trip. Think about other uses you have for your money. No real purchase will take place, but we ask that you imagine you are making real choices that would result in you purchasing the products you select*.”

Participants made choices in five categories: ready-to-eat breakfast cereals, bread, crackers, pasta/dry goods (which included rice, legumes, and other grains), and produce. In each category, participants were instructed to “*Please select one item that you would like to purchase. You may also indicate that you would not purchase any of these items if you do not find one that you are interested in purchasing*.” These categories were presented to participants in a random order. Each food category featured 33 unique products. Reflecting many online food retail environments, participants had the option to view a subset of the available products or to view all available products. Every product category featured three subsets, each of which contained 11—one-third—of the products.

The subsets of products in each product category were organized based on real-world retailers' practices. For more highly processed products, such as cereals, breads, and crackers, this resulted in subsets with significant differences in nutrient levels between sets but limited variation in nutrient content within sets. These subsets feature 1) zero, 2) one, and 3) two or three stars. For instance, the cereal category in both physical and online retail environments frequently features cereals clustered into groups such as “Kids' Cereals,” “Family Favorites,” and “Healthy Options.” Less processed product categories, such as *Produce* and *Dry Goods and Pasta* are not separated into subsets in a way that creates large differences from one subset to another and little variation within a set. The category, *Dry Goods and Pasta*, was separated into subsets of *Pasta, Rice*, and *Legumes and Other Grains*, based on real-world retailer segmentation. The pasta category included whole wheat pasta varieties, which are rich in dietary fiber, but also pasta made from refined flour and gluten-free pastas, both of which have poorer nutritional profiles. On the other hand, products in the *Legumes and Other Grains* category are uniformly of high nutritional quality (nearly all 3 stars on the Guiding Stars scale). The products included in each category, the subset each product belonged to within the category, and nutritional information for the product, including Guiding Stars, are presented in Supplementary Materials Tables 1–5. To analyze the choice of consideration set, each set within a food product category was ranked based on the mean nutritional quality of the products in each set from 1 (lowest mean quality) to 4 (highest mean quality).

For the products that were divided into subsets that differed by nutrition content (cereals, breads, and crackers), the subsets were named based on examples of the products contained in each subset: “Cereals such as Frosted Flakes, Froot Loops, Reese's Puffs” rather than “Kids' Cereals.” This decision was meant to avoid potential social desirability biases that might result from an adult not wanting to choose a cereal from a subset called “Kids' Cereals” when they know that those choices will be reviewed by a researcher (33).

Participants selected the set of products they wanted to view—all, or one of the three subsets—and then viewed the products in the selected set for each category. Participants chose one of the products in the selected set; however, they could also indicate that they would not purchase any of the available products. Thus, participants would choose at most one item in each product category.

### Use of Nutrition Information During Food Choice, Consideration of Future Health, and Demographic Questions

Information about key nutritional attributes that are featured on nutrition facts panels—calories, fat, sodium, sugars, and dietary fiber—were listed below each product. Participants were able to view this information without taking any additional actions. After completing the food choices in the five food categories, participants were asked to indicate nutrition information they had considered during food choice for each food category. These questions were asked in a check-all-that-apply format for each food category, leading to a binary measure for each nutrient in every food category. Participants then responded to the question about broader considerations during choice. This question, “*In general, when you were making food choices in the survey, did you think about*: ?” included six potential responses. The key response of interest was “*Impacts the foods might have on your/your family's health in the future*;” other responses were included as decoy options to diminish experimenter demand or social desirability bias effects. Direct elicitation of a variable of interest after collection of other data critical to the analysis—the food choices in this case—is used when asking during the process might change the decision-making process (34).

Finally, participants responded to standard demographics questions, such as gender, age, education, and income. Only data from individuals who made food choices in every category are included so that the outcome variable—average Guiding Stars in the bundle of five (three processed) products chosen—is comparable across all individuals.

### Data and Analysis

First, summary statistics for demographic characteristics and whether the respondent considered future health impacts of food choice are reported. The demographic variables reported are 1) whether the respondent is female, 2) age of the respondent, 3) education level of the respondent, and 4) household income in 2020. The variable, *Female*, is binary (=1 if the respondent was female; =0 otherwise). The respondents' age, education, and household income were collected in ranges and converted to a numeric variable. Age and household income were converted to a numeric variable using the midpoint of the range the individual selected. Education was converted to a numeric variable based on the time to complete the highest level of education the individual had attained.

#### Analysis of Relationship Between Consideration of Future Health and Choice Variables

Next, the relationship between consideration of future health impacts and choices in the experiment was examined, using only observations from participants in the control condition (*n* = 2309). The Guiding Stars rating of the product was used as a measure of the overall nutritional quality of food products chosen for the analyses. Note, however, that Guiding Stars information was not provided to participants—it was used solely for analysis. The Guiding Stars rating system uses the nutrient levels of food products to generate a score that classifies foods into one of four categories, ranging from zero (lowest nutritional quality) to three (highest nutritional quality) stars (www.guidingstars.com). First, the effect of consideration of future health impacts on the number of Guiding Stars received by the products chosen in the food choice task for 1) all food products and 2) processed foods (ready-to-eat breakfast cereals, bread, and crackers) was estimated using linear regression analysis. In a second version of the regression, demographic variables, including gender, education, income, and age, that may affect respondents' tendencies to make healthier choices were added to test the robustness of the results.

Next, the impact of consideration of future health impacts on choice process variables was examined: the nutritional quality of the set of products the participants considered during the choice process and the use of nutrition information during choice for all products and for processed products. For the set of products the individual considers, the average rank of the set of the products (for all products, the summed rank of all five categories divided by five and the summed rank of cereal, bread, and cracker categories divided by three for the processed food categories) the respondent selected to view during choice was used to identify a relationship with consideration of future health impacts; a second version of the regression with demographic variables is reported to test the robustness of the findings. To evaluate the use of nutrition information, a linear regression model, with the average number of nutrients considered across the five (three) product categories as the dependent variable and consideration of future health impacts as the explanatory variable, is estimated. In a second version of the regression, demographic variables are added to test the robustness of the estimates in the first regression. Additionally, the three sets of analyses discussed above were repeated using ordinal logistic regression. The results in terms of sign and significance of the variables are consistent between the two approaches. The linear regression results are reported for ease of interpretation.

#### Impact of a Health Prompt Message on Consideration of Future Health and Choice Outcomes

Next, the impact of a simple health prompt message on consideration of future health impacts and on the nutritional quality of foods chosen is examined. Due to an error in the survey design, participants were not required to spend a minimum amount of time on any of the pages in the online survey. However, the time a participant spent on each page was tracked, which is used to estimate the impact of a health message on consideration of future health impacts during food choice. The estimation strategy makes use of the fact that the page preceding the health message was a set of general instructions about the experiment, which all participants viewed. The median time spent on the prompt message, which only participants in the prompt condition were exposed to, was 3.0 sec. The median time spent on the general instructions page was also 3.0 sec, and the relationship between time spent on the instructions and the time spent on the prompt page was highly significant (*p* <0.001), making time spent on the instructions page a good variable to control for potential differences that may exist in participants who spend more or less time reading text. For instance, individuals who voluntarily spend more time reading text may be more likely to consider broader implications of the choices they face than people who prefer not to spend time reading text if they are not required to. By using the time spent on the instruction page in addition to time spent on the fiber message, the relationship between a general inclination to voluntarily read text and consider future health implications is controlled for. Two cut-off points for time spent on the instructions and prompt pages are used in different versions of the analysis. The first version of the analysis uses the median time spent on both pages: 3 sec. A dummy variable that takes the value of 1 for any participant who spent the median amount of time or longer on the page was created. The second cut-off point used is 10 sec, which is approximately the amount of time that that it would take an individual to quickly read the prompt text based on tests run by the researcher. The prompt message read, “*How can dietary fiber help you reach your health goals? While some benefits of fiber consumption are well known, dietary fiber has a number of surprising benefits. Benefits that are not widely known include that dietary fiber: 1. Reduces energy intake (by, for example, promoting feelings of fullness), which helps with weight loss; 2. Lowers blood pressure; 3. Increases absorption of important minerals; 4. Lowers blood glucose; 5. Lowers cholesterol levels. Choosing products with higher dietary fiber can help you meet your health goals!*”

To examine the impact of exposure to a health prompt on consideration of future health impacts of food choice, a logistic regression of the consideration of future impacts (dependent variable) on the prompt (at 3 and 10-sec levels in separate regressions), time spent on general instructions (3 and 10-sec levels), and demographic characteristics was estimated to generate adjusted odds ratios and 95% confidence intervals. Finally, a linear regression analysis estimates the effect of consideration of future health impacts on Guiding Stars ratings of choices for the full sample while accounting for the time spent on prompt and instructions variables (with interaction terms between consideration of future health impact and prompt and instruction variables to allow the estimated impact of consideration to vary by condition).

R statistical software was used to conduct the analyses (35). The research was approved by the university's institutional review board. Results with *p*-values <0.05 are considered to be significant.

## Results

Summary statistics of the participants' demographic characteristics are reported in Table 1, along with the proportion of participants who reported considering the future health impacts of foods during the choice process. Just over half (52.6%) of participants were female. Participants were approximately 45 years of age (1.6% of participants declined to report their age). Approximately half of participants (50.4%) had received at least a college level education. Average household income (in 2020) was just over $77,000 (5.2% of participants declined to report income). Our respondents are similar to US demographic characteristics for sex, age, and income. Females comprise 51% of the US population, 16.5% of the US population is over 65 years of age (compared to 16.4% of our sample), and the median income of US households is $62,843. In our sample, income was collected in ranges (and converted to the midpoint of the range for regression analysis); the median household income fell between $60,000-79,999. On the other hand, the sample has a significantly higher level of education—approximately 33% of the US population (≥25 years) has a bachelor's degree, while over half of our participants did. US demographic characteristics are drawn from the US Census Bureau's Quick Facts tool (36). Importantly, few participants—only 31%—reported considering future health impacts of foods during choice.

**Table 1:** Summary statistics on consideration of future health impacts during food choice and demographic characteristics of US respondents.

**Variable**	**%/Mean (SD)**
Consideration of future health (%)	31.1
Female (%)	52.6
Age (years)	44.3 (16.0)
Education (years)	15.1 (2.6)
Income ($1000s)	77.6 (55.5)
N	4622

*Consideration of future health is reported only for participants in the control condition. Individuals who preferred not to respond to the questions about age, education, and income were omitted from the calculation of these variables. There were no significant differences in female, age, education, or income between conditions*.

### Impact of Active Consideration of Future Health on Nutritional Quality

The analysis of the relationship between active consideration of future health impacts and the number of Guiding Stars per category in the products selected in the food choice task is reported in Table 2 for all products (columns 1 and 2) and processed products (columns 3 and 4).

**Table 2:** The impact of consideration of future health on average guiding stars selected per product in control condition.

	**All products**		**Processed products**	
	**(I)**	**(II)**	**(III)**	**(IV)**
	**Coefficient**	**Coefficient**	**Coefficient**	**Coefficient**
	**(SE)**	**(SE)**	**(SE)**	**(SE)**
Intercept	1.330^***^ (0.013)	0.832^***^ (0.048)	0.810^***^ (0.015)	−0.033 (0.093)
Consideration of Future Health Impacts	0.124^***^ (0.023)	0.124^***^ (0.022)	0.206^***^ (0.028)	0.194^***^ (0.028)
Female		0.034 (0.022)		0.008 (0.028)
Age		0.006^***^ (0.001)		0.006^***^ (0.001)
Income		0.0005^*^ (0.0002)		0.001^***^ (0.000)
Education		0.022^***^ (0.005)		0.034^***^ (0.006)
Adjusted R2	0.015	0.080	0.027	0.097
*N*	1863	1783	1964	1873

*** *= p-value < 0.001*;

* *= p—value < 0.05. Data from control condition only*.

Individuals who considered future health consequences chose a basket of products with significantly more Guiding Stars than those who did not consider future health consequences both for all products and for processed products. The estimated impact of future health consideration is larger for the subset of processed products—approximately 0.2 additional GS for processed products vs. 0.12 for all products. The estimated coefficient on consideration of future impacts means that someone who considered the future impacts of foods would, on average, choose a product with an additional GS in the course of selecting a basket of five processed products relative to an individual who did not consider future health impacts. The larger estimated impact of consideration of future health impacts among processed products likely reflects lower average Guiding Stars ratings for the processed food items available to choose among (mean GS per product for processed products = 1.03 to 1.09) than for the produce items (mean GS per product = 2.6) or pasta, grain, and legume items (mean GS per product = 1.9). The sets of items with product characteristics are available online as Supplementary Materials. The estimated effect of consideration of future health impacts is robust to the inclusion of demographic control variables. However, many demographic variables are significantly related to nutritional quality of food choices. Age, income, and education are all positively related to nutritional quality of food choices. Both age and education are highly significant (*p* <0.001). An additional year of age is associated with the choice of products with an extra 0.006 GSs, while an additional year of education leads to over 0.05 additional GSs. An additional $1000 of household income is associated with just under 0.01 more GSs. Importantly, consideration of future health impacts has a markedly higher impact on GSs than any of the demographic variables.

### Choice Process Variables: Choice Set Rank and Use of Nutrition Information

The relationship between consideration of future health impacts and choice set rank is reported in Table 3. Consideration of future health impacts is again highly significant (*p* <0.001) and robust to the inclusion of demographic variables. Individuals who consider the future health impacts of food choice selected an average of one higher nutritional quality consideration set across the five food categories. Age and education are also statistically significant for both all products and processed products. Like in the analysis of GSs, consideration of future health impacts has a much larger impact on increasing the rank of the nutritional quality of consideration sets than demographic control variables.

**Table 3:** Impact of future consideration of health on nutritional quality-based rank of the set of products participants chose to view in the control condition.

	**All products**		**Processed products**	
	**(I)**	**(II)**	**(III)**	**(IV)**
	**Coefficient**	**Coefficient**	**Coefficient**	**Coefficient**
	**(SE)**	**(SE)**	**(SE)**	**(SE)**
Intercept	2.19^***^ (0.01)	1.56^***^ (0.09)	2.25^***^ (0.02)	1.42^***^ (0.11)
Consideration of Future Health Impacts	0.19^***^ (0.03)	0.20^***^ (0.03)	0.24^***^ (0.03)	0.24^***^ (0.03)
Female		0.04 0.03		0.04 (0.03)
Age		0.006^***^ (0.001)		0.005^***^ (0.001)
Income		0.0003 (0.0002)		0.0007^*^ (0.0003)
Education		0.021^***^ (0.005)		0.034^***^ (0.007)
Adjusted R2	0.021	0.060	0.021	0.056
N	2309	2150	2309	2150

*** *= p-value < 0.001*,

* *= p-value < 0.05. Data from control condition only*.

Table 4 reports the results of the analysis of use of nutrition information and consideration of future health impacts. Consideration of future health impacts is significantly and positively related to the use of nutrition information during food choice (*p* <0.001). Individuals who considered future health impacts of food during choice made use of approximately one more piece of nutrition information than those who did not consider future health impacts per food category. The result is again robust to the inclusion of demographic characteristics and whether examining all products or the subset of processed products.

**Table 4:** The impact of future health consideration on use of nutrition information during food choice in the control condition.

	**All products**		**Processed products**	
	**(I)**	**(II)**	**(III)**	**(IV)**
	**Coefficient**	**Coefficient**	**Coefficient**	**Coefficient**
	**(SE)**	**(SE)**	**(SE)**	**(SE)**
Intercept	0.764^***^ (0.028)	0.346^*^ (0.165)	0.832^***^ (0.029)	0.224 (0.174)
Consideration of Future Health Impacts	0.979^***^ (0.049)	0.886^***^ (0.050)	1.060^***^ (0.051)	0.969^***^ (0.052)
Female		−0.174^***^ (0.048)		−0.153*^*^ (0.051)
Age		−0.010^***^ (0.001)		−0.008^***^ (0.002)
Income		0.001 (0.001)		0.001 (0.001)
Education		0.064^***^ (0.011)		0.068^***^ (0.011)
Adjusted R2	0.147	0.197	0.155	0.192
N	2309	2150	2309	2150

*** *= p-value < 0.001*,

* *= p-value < 0.05. Data from control condition only*.

Female, age, and education are also significant variables in explaining the use of nutrition information. Both being female and being older are associated with a decrease in the number of pieces of nutrition information used during choice, while more education increases the use of nutrition information.

### Effect of Prompt on Consideration of Future Health Impacts of Food Choice

The next analysis examines whether exposure to the prompt increases consideration of future health impacts of food choice. The results (adjusted odds ratios and 95% confidence intervals) of the series of regressions are presented in Table 5.

**Table 5:** Adjusted odds ratios (aOR) and 95% confidence intervals (95%CI) from logistic regression of fiber prompt message on consideration of future health impacts of foods during food choice.

	**3 sec**		**10 sec**	
	**aOR**	**aOR**	**aOR**	**aOR**
	**(95%CI)**	**(95%CI)**	**(95%CI)**	**(95%CI)**
Prompt	1.18 (1.02, 1.36)	1.30 (1.11, 1.51)	1.39 (1.15, 1.68)	1.58 (1.29, 1.92)
Instruction (Control)	1.07 (0.94, 1.22)	1.11 (0.97, 1.26)	1.08 (0.93, 1.26)	1.10 (0.94, 1.29)
Female		1.00 (0.88, 1.15)		1.01 (0.88, 1.15)
Age		0.99 (0.98, 0.99)		0.99 (0.98, 0.99)
Education		1.11 (1.07, 1.14)		1.11 (1.07, 1.14)
Income		1.001 (1.0003, 1.003)		1.001 (1.0003, 1.003)
Intercept	0.43 (0.39, 0.47)	0.14 (0.09, 0.22)	0.44 (0.41, 0.47)	0.15 (0.09, 0.23)

*Data from control and prompt conditions*.

Exposure to the prompt for 3 and 10-sec thresholds significantly increases the likelihood that participants consider the future impacts of food choices across all models compared to those who were not exposed to the prompts (for the threshold time). Individuals who spent at least 3 sec on the prompt page were 1.2 times more likely to consider the future health impacts of foods during choice without demographic controls and 1.3 times more likely taking demographic controls into account. Participants who spent 10 or more seconds on the prompt were 1.4 times more likely to consider future health impacts (no demographic controls) to nearly 1.6 times more likely (with demographic controls). The variable, *Instruction (Control)*, that accounts for individual differences in reading information is not significant in any of the analyses, though the effect is consistently estimated across regressions. Demographic characteristics capturing age, education, and income are significant, though the adjusted odds ratios for age and income are small. An additional year of age decreases the likelihood of considering future health impacts by 0.99, while an additional $1000 of household income increases the likelihood of considering future health impacts by 1.001 times. Education has a larger impact. An additional year of education increases the likelihood of considering future health impacts during food choice by 1.11 times.

Finally, the results of the regression analyzing the impact of consideration of future health impacts of food choice interacted with attention to the fiber prompt message and general instructions (at the 10-sec level) are presented in Table 6. The estimated effect of considering future health impacts on the average number of Guiding Stars chosen falls within the same range (~ 0.10–0.12 for all products; 0.19–0.20 for the subset of processed products) for the whole sample as for the control group. Additionally, exposure to the prompt (10 sec) significantly increased the number of Guiding Stars chosen. Interaction effects between prompt and consideration of future health impacts were not significant. These findings suggest that consideration of future health leads to healthier choices.

**Table 6:** The impact of consideration of future health impacts and fiber prompt (10 second exposure) on average guiding stars of foods selected in food choice experiment.

	**All products**		**Processed products**	
	**(I)**	**(II)**	**(III)**	**(IV)**
	**Coefficient**	**Coefficient**	**Coefficient**	**Coefficient**
	**(SE)**	**(SE)**	**(SE)**	**(SE)**
Intercept	1.332^***^ (0.011)	0.768^***^ (0.053)	0.807^***^ (0.013)	0.108 (0.065)
Consideration of Future Health Impacts (CFHI)	0.114^***^ (0.018)	0.107^***^ (0.018)	0.209^***^ (0.023)	0.192^***^ (0.023)
Prompt (10 sec.)	0.089^**^ (0.031)	0.077^*^ (0.031)	0.114^**^ (0.037)	0.109^**^ (0.037)
Instructions (10 sec.)	0.009 (0.023)	0.003 (0.023)	0.002 (0.028)	0.005 (0.028)
CFHI*prompt	0.071 (0.049)	0.041 (0.048)	0.086 (0.059)	0.052 (0.059)
CFHI*instructions	0.065 (0.039)	0.071 (0.048)	0.071 (0.048)	0.072 (0.048)
Demographics	No	Yes	No	Yes
Adjusted R2	0.031	0.082	0.046	0.096
N	3931	3747	3931	3747

*** *= p-value < 0.001*,

** *= p-value < 0.01*;

* *= p-value < 0.05. Data from control and prompt conditions*.

## Discussion

While previous research has documented robust relationships between a tendency to consider the future and positive outcomes in important domains such as health behaviors, financial decision-making, and education choices, the tools used to measure future orientation were intended to capture a static trait of individuals, which appears to be accurate in the mid-term (8). Research indicating context-specific influences on cognitive processes suggest that while there may be stable tendencies to consider the future during decision-making, there is likely temporal variation that depends on a variety of factors (10, 12, 14).

In this paper, the relationship between choice-specific consideration of future health impacts and higher nutritional quality of foods selected during a choice task undertaken by a large sample of respondents that is nationally representative of key demographic characteristics, including gender, age, and income was examined. The research shows that active consideration of future health impacts during the choice process leads to the choice of foods that are of higher nutritional quality. The findings also identify a positive relationship between consideration of future health impacts and behaviors that support healthier food choices in complex choice environments. Specifically, individuals who consider future health impacts direct their attention to healthier sets of foods during food choice and use significantly more nutrition information during the choice process. Finally, attention to a short prompt message highlighting specific health benefits of fiber increases consideration of future health impacts during food choice (while controlling for attention to general survey instructions and demographic characteristics).

Choice instance-specific variation in consideration of future impacts of choices has different implications for policies that are intended to promote healthy choices—whether for foods or in other domains—than if consideration of the future is a stable trait. Perhaps the most important difference is that choice-specific variation in future consideration permits strategies that seek to prompt people to consider the future during the choice process. In a working paper, Urminsky and Goswami (37) find that the primary effect of calorie labeling is to remind shoppers to think about the nutritional quality of foods rather than providing functional information. Reminders sent to gym members increase gym attendance, with an effect that lasts beyond the end of the intervention (38, 39). Recent work on primes or prompts encountered by individuals in food choice and physical activity environments finds increases in healthy behaviors, suggesting that these interventions draw attention to the health attributes of choices (16, 17, 28, 40–42). In this work, results show that participants who spend at least 3 sec on the prompt message are significantly more likely to consider future health impacts and that the effect size of the prompt message increases as participants spend more time processing the information. Tests by the researcher of the amount of time needed to read the prompt message show that it takes approximately 10 sec. Participants who spent 3–5 sec on the page may have been reminded to think about health implications in response to the mention of fiber, but those who spent the time to read the message encountered text enumerating multiple health benefits of fiber occurring over different time scales, which may have more directly prompted consideration of the future health implications of food choices. Analysis of the nutritional quality of choices does not detect significant interaction effects between exposure to the prompt and consideration of future health impacts, while each is separately independent.

This work does have some limitations that need to be addressed in future work. First, the choice task featured hypothetical choices. The decision to use a hypothetical choice task was made in order to permit the collection of data from a large (*n* = 4622) sample that is nationally representative according to important demographic characteristics, such as gender, age, and income; however, there are frequently concerns about decisions that are made under hypothetical conditions. This is potentially less of a concern with food choices, as food choices are highly habitual for most people (26). Nevertheless, a cheap-talk script that directed respondents to consider other ways in which they could spend their money when considering whether they would purchase a food item or not was included. Cheap-talk scripts have been shown to reduce hypothetical biases in economic valuation studies (32).

Second, the key question in the research asked participants whether they had considered future health impacts during the choice process. While this captures people who actively considered health while making choices among the available food products, it does not allow for individuals who did not actively consider the future health impacts of the foods in this choice scenario because they previously considered the health impacts of food and now those choices were simply habitual. If individuals with previously established healthier dietary patterns comprise a non-negligible percentage of the sample, this would mean that our results may be a conservative estimate of the impact of considering future health impacts during food choice.

Third, the prompt experiment was not carried out according to our intention, resulting in a situation in which not all participants assigned to the health message intervention were truly exposed to the message. While the results show significant, robust evidence that attention to the prompt increases consideration of long-term health implications of food choice, the implications are not as broad as they potentially might have been. By controlling for a tendency to pay attention to general instructions, the estimated impact of attention to the prompt is not simply detecting a correlation between attention to details and thinking more broadly about the implications of choices. However, the results do not provide evidence about how individuals who did not attend to the prompt message would have responded had they been required to read the message. The realized experiment more accurately reflects reality—individuals have some ability to ignore or pay attention to information in the real world—but it would be of interest to have a baseline assessment of the impact of health-related prompts on consideration of future outcomes.

An additional concern is the influence of social desirability bias (33). Social desirability bias refers to an inclination to alter one's responses to questions based on the perception that one response is more socially acceptable. The respondents completed the research online and were recruited and guided through the research process by an intermediary, making the respondents fully anonymous to the researcher. This should reduce the impact of social desirability bias, though it cannot be ruled out. However, if social desirability bias is present in reporting consideration of future health impacts, it would attenuate the estimated impact of the variable, resulting in a conservative estimate.

A final limitation is that the adjusted R2 was low for models examining the impact of future health consideration on GS of chosen products. This is due to the complexity of the choice environment and the ability of participants to direct their attention to subsets of products and information. Participants selected from a set of 33 products per product category. However, they could, if they wanted, choose to view subsets of the available products and use nutrition information (or not) during food choice, meaning that—depending on the choices about the set of products and information to consider—one participant might face a very different set of products with different nutritional information when making a choice than another participant. The results in Tables 3, 4 show that those participants who considered the future health impacts of food during the choice process chose to view sets of products with higher nutritional ranking, based on the average GS rating of the products in that set (Table 3) and used more pieces of nutrition information during food choice (Table 4) than participants who did not consider future health impacts. Selecting a healthier subset of products to view affects the GS rating of the products chosen because products were categorized into subsets based on GS ratings. Thus, the inclusion of the consideration set variable would explain a lot of variation in GS of products chosen. To estimate the impact of the consideration of future health impacts on the GS of products chosen, the choice process variables related to the set of products that participants chose to view and the nutrition information used were omitted from the regressions. Including those two variables in the regression analysis increases the adjusted R2 of, for instance, regression IV in Table 2 from 0.097 to 0.614. Consideration of future health impacts is still significant in this case, but it is reduced markedly—from 0.194 to 0.042—because the other choice process variables related to the set of products considered and the nutrition information used, which are also affected by consideration of future health impacts, contribute a marked amount of explanatory power.

There is growing evidence that point-of-decision interventions can help people make healthier choices (16, 17, 40, 41). This paper reports evidence that some of the effects of these interventions may result from prompting consideration of long-term impacts of those choices in the specific choice scenario. Future research will build on this research to refine evidence of the potential to prompt individuals to consider long-term impacts of their choices and to explore the impact of those prompts.

## Data Availability Statement

The raw data supporting the conclusions of this article will be made available by the authors, without undue reservation.

## Ethics Statement

The studies involving human participants were reviewed and approved by University of Nebraska-Lincoln Institutional Review Board. The patients/participants provided their written informed consent to participate in this study.

## Author Contributions

CG conducted the research, analyzed the data, wrote and edited the manuscript, and approved the submitted version.

## Funding

This research was conducted with financial support provided by the National Institutes of Health (NIH) grant P20GM104320.

## Conflict of Interest

The author declares that the research was conducted in the absence of any commercial or financial relationships that could be construed as a potential conflict of interest.

## Publisher's Note

All claims expressed in this article are solely those of the authors and do not necessarily represent those of their affiliated organizations, or those of the publisher, the editors and the reviewers. Any product that may be evaluated in this article, or claim that may be made by its manufacturer, is not guaranteed or endorsed by the publisher.
